# Unveiling the Genetic Landscape of Feed Efficiency in Holstein Dairy Cows: Insights into Heritability, Genetic Markers, and Pathways via Meta-Analysis

**DOI:** 10.1093/jas/skae040

**Published:** 2024-02-13

**Authors:** Wentao Jiang, Mark H Mooney, Masoud Shirali

**Affiliations:** Institute for Global Food Security, School of Biological Sciences, Queen’s University Belfast, Belfast, BT9 5DL, UK; Agri-Food and Biosciences Institute, Large Park, Hillsborough, BT26 6DR, UK; Institute for Global Food Security, School of Biological Sciences, Queen’s University Belfast, Belfast, BT9 5DL, UK; Institute for Global Food Security, School of Biological Sciences, Queen’s University Belfast, Belfast, BT9 5DL, UK; Agri-Food and Biosciences Institute, Large Park, Hillsborough, BT26 6DR, UK

**Keywords:** feed efficiency, Holstein dairy cows, heritability, genetic factors, pathway analysis

## Abstract

Improving the feeding efficiency of dairy cows is a key component to improve the utilization of land resources and meet the demand for high-quality protein. Advances in genomic methods and omics techniques have made it possible to breed more efficient dairy cows through genomic selection. The aim of this review is to obtain a comprehensive understanding of the biological background of feed efficiency (**FE**) complex traits in purebred Holstein dairy cows including heritability estimate, and genetic markers, genes, and pathways participating in FE regulation mechanism. Through a literature search, we systematically reviewed the heritability estimation, molecular genetic markers, genes, biomarkers, and pathways of traits related to feeding efficiency in Holstein dairy cows. A meta-analysis based on a random-effects model was performed to combine reported heritability estimates of FE complex. The heritability of residual feed intake, dry matter intake, and energy balance was 0.20, 0.34, and 0.22, respectively, which proved that it was reasonable to include the related traits in the selection breeding program. For molecular genetic markers, a total of 13 single-nucleotide polymorphisms and copy number variance loci, associated genes, and functions were reported to be significant across populations. A total of 169 reported candidate genes were summarized on a large scale, using a higher threshold (adjusted *P* value < 0.05). Then, the subsequent pathway enrichment of these genes was performed. The important genes reported in the articles were included in a gene list and the gene list was enriched by gene ontology (**GO**):biological process (**BP**), and Kyoto Encyclopedia of Genes and Genomes (**KEGG**) pathways analysis. Three GO:BP terms and four KEGG terms were statistically significant, which mainly focused on adenosine triphosphate (ATP) synthesis, electron transport chain, and OXPHOS pathway. Among these pathways, involved genes such as ATP5MC2, NDUFA, COX7A2, UQCR, and MMP are particularly important as they were previously reported. Twenty-nine reported biological mechanisms along with involved genes were explained mainly by four biological pathways (insulin-like growth factor axis, lipid metabolism, oxidative phosphorylation pathways, tryptophan metabolism). The information from this study will be useful for future studies of genomic selection breeding and genetic structures influencing animal FE. A better understanding of the underlying biological mechanisms would be beneficial, particularly as it might address genetic antagonism.

## Teaser

This study aimed to summarize existing knowledge on reported heritability, genetic markers, genes, and biological mechanisms of feed efficiency complex in purebred Holstein cows. Then a meta-analysis was subsequently performed to improve the existing understanding of the biological background of FE.

## Introduction

Feed costs account for a large portion of variable and total costs associated with the farm, up to 60% (Connor, 2015). Breeding highly-efficient animals can improve farm profitability and reduce the environmental impact of animal production (Lovendahll, 2018). With the development of genomic methods and omics techniques, it is feasible to select high-yielding cows by feed efficiency (**FE**) index (Madilindi et al., 2022b). FE traits are typical complex traits controlled by multiple genes, and a single gene has little influence on the traits (Madilindi et al., 2022b). Representative FE traits include dry matter intake (**DMI**), energy balance (**EB**), residual feed intake (**RFI**), gross feed efficiency (**GFE**), residual efficiency intake (**REI**), etc. In animal breeding, narrow-sense heritability refers to the fraction of phenotypic variance that can be attributed to variation in the additive effects of genes (Englishby et al., 2016), while broad-sense heritability refers to the proportion of phenotypic trait changes resulting from genetic variation between animals (Wray and Visscher, 2008). It is usually estimated by comparing phenotypic differences and phenotypic records of individual animals with pedigree information (Yang et al., 2017). Accurate genetic assessment is a prerequisite for genomic selection, which relies on a large amount of genomic information and animal phenotypic recording.

Genomic selection usually captures the effects of causative mutations on certain traits by screening thousands of mutation sites throughout the genome. These causative mutations captured by genomic selection can be tagged by polymorphisms. The effect of the causative mutation is usually located among genotyped single-nucleotide polymorphisms (**SNPs**). The advent of commercially available high-density oligonucleotide SNP arrays/SNP chips allows the simultaneous detection of large numbers of SNPs, making genomic selection possible (Eggen, 2012). In addition, the declining cost of sequencing and the development of multi-omics techniques have brought large amounts of data and detailed genomic information to genomic selection. Possible loci influencing FE as well as mechanisms of related biological pathways are emerging from genome-wide association studies (**GWAS**) (Lu et al., 2020), transcriptomic studies (Shi et al., 2018), metabolomics studies (Martin et al., 2021), and candidate gene studies (Delosière et al., 2019). These findings could be used to develop precise breeding programs and possibly address genetic antagonism (Berry et al., 2014b).

At present, many studies have reported the heritability estimation and genetic factors of FE complex in dairy cows. Here, we focused on purebred Holstein cows to eliminate the bias caused by different breeds. Holstein dairy cow is one of the most representative breeds; Holestein cows have the highest mean milk yield among all commercial dairy breeds (VanRaden and Sanders, 2003) and were greatly improved through genomic selection during the last half of the 20th century (Rodríguez-Bermúdez et al., 2019). Therefore, this study has sought to summarize existing knowledge on reported heritability, genetic markers, genes, and biological mechanisms of FE complex in purebred Holstein cows. Subsequent meta-analyses of heritability estimates and reported candidate genes were independently performed to improve the existing understanding of the biological background of FE.

## Materials and Methods

### Search strategy

A search protocol was established based on the Preferred Reporting Items for the Systematic Reviews and Meta-Analyses (PRISMA) framework (Page et al., 2021). For Population, Exposure, and Outcome (PEO) components of PRISMA framework, the population was defined as “Holstein dairy cows”, with an exposure of “genetic factors” and the outcome of “traits”. Each PEO component was connected by the Boolean operator “AND”. The keywords in each category were connected by the Boolean operator “OR”. At least one keyword of each PEO component could be returned by this search algorithm. Keywords of each category were discussed and agreed by the authors, and a total of 720 combinations were used as queries for searches (Table 1). The publications used in the systematic review were searched using the Web of Science Core Collection web search engine. Literature published dates ranged from January 2000 to June 2023.

**Table 1.** Terms of population, exposure, outcome (PEO) framework used in literature search

Population (Holstein dairy cows)	Exposure (genetical factors)	Outcome (traits)
“Dairy cow” “Dairy Cattle” Heifer Holstein Friesian	Pathway QTL SNP CNV Gene Genetic* GWAS Genomic* Heritab* Transcriptom* RNA* marker biomarker variant “Network analysis” expression	“Feed efficiency” “Feeding efficiency” “Residual feed intake” “Residual solid production” “Energy balance” “Residual Nitrogen intake” “Nitrogen use efficiency”

### Literature selection

Two researchers conducted searches and assessments concurrently and independently. A final literature list was agreed upon after a discussion of different selected studies. Studies were excluded for three main categories: “No pure Holstein dairy cow”, where the subject was on other species, breeds, crossbreeds, beef or bull; “No FE traits”, where the study primary focus related to other traits (e.g., production, nutrition, economic, emission, rumen microbe, fertility); “Animal health and welfare”, which focused on animal disease or improving fertility. Some studies that have no genomic focus (e.g., feeding system, diet suggestion, FE traits prediction/measurement) were excluded.

### Integration of results

The articles selected by the literature were divided into three groups according to their content: “Heritability”, “Genetic markers and genes”, and “Pathways”. For “Heritability” articles, information including phenotype, number of animals with phenotype records, and estimated heritability with standard error was tabulated. For “Genetic markers and genes” articles, only genetic markers and genes that met statistical significance requirements were included. Considering most studies adopted multiple comparisons and multiple test methods, a false discovery rate (**FDR**)-adjusted *P* value (or *Q* value) of 0.05 was considered statistically significant. All the genetic markers reported in the articles were annotated. Initially, statistically significant genes from three sources were annotated: 1) candidate genes mapped around reported genetic markers within 1 Mb interval (500 kb upstream and 500 kb downstream), 2) mapped genes in the associated window, and 3) differentially expressed genes. Genomic coordinates for genetic markers and genes were converted using the assembly ARS-UCD1.2 of the bovine genomes as a reference. Then, the list of these genes was applied for pathway enrichment. For “Pathways”, all reported pathways in articles were included.

### Meta-analysis

#### Meta-analysis of heritability estimates

A random-effects model based on comprehensive meta-analysis was applied to perform a meta-analysis of FE heritability estimates from studies using the R package meta (Borenstein et al., 2010). The random-effects model is shown as follows:


$$\begin{array}{l} Y_{i}\;=\mu +\;\xi_{i}+\;\varepsilon_{i} \end{array}$$

where ξ_*i*_ is the difference between the grand mean (µ) and the true mean (θ_*i*_) for study *i* (ξ_*i*_ = θ_*i*_ − µ) and ε_*i*_ is the difference between the true mean for study *i* (θ_*i*_) and the observed mean (*Y*_*i*_) for study *i* (ε_*i*_ = *Y*_*i*_ − θ_*i*_). Thus, the total variance of the random-effects model consists of two parts: the intra-study variance (*V*_*i*_) and the inter-study variance (*T*^2^).

Pooled estimates of heritability were considered significant at *P* value ≤ 0.05. The results of the meta-analysis are presented in forest plots graphically. In the forest plots, the square size represents the weight of the study. The larger the square size indicates the more weight of the study on the mean effect size. The horizontal line represents a 95% confidence interval (95% CI).

Heterogeneity between studies was assessed by the *I*^2^ index and Chi-square (χ^2^) test (Borenstein et al., 2010). The calculation of *I*^2^ index is shown as follows:


$$\begin{array}{l} I^{2}\left(\%\right)\;=\frac{\chi^{2}-(n-1)}{Q}\times \;100 \end{array}$$

where χ^2^ is the chi-square statistic value for heterogeneity and *n* is the number of studies. The Chi-square is calculated as follows:


$$\begin{array}{l} \chi^{2}=\overset{n}{\underset{i=1}{\sum }}\;\;W_{i}{\left(\theta_{i}-\theta \right)}^{2} \end{array}$$

where *W*_*i*_ is the weight parameter estimate in the *i*th study and *n* is the number of studies.

An estimated value of *I*^2^ greater than 50% indicates high heterogeneity (Nakagawa and Santos, 2012).

#### Meta-analysis of gene list (pathway enrichment)

All genes fulfilling the criteria described above were applied for meta-analysis. Initially, positions and annotations of genes were converted by the bovine genome browser—Ensembl (Cunningham et al., 2021). Gene list was then enriched by two functional enrichment analysis tools: gProfiler (https://biit.cs.ut.ee/gprofiler/gost) and DAVID (https://david.ncifcrf.gov/summary.jsp). Functional information was retrieved from the following sources: gene ontology (**GO**) terms for biological process (**BP**), and Kyoto Encyclopedia of Genes and Genomes (**KEGG**) cellular component. An FDR-adjusted *P* value (or *Q* value) of 0.05 was considered statistically significant.

## Results and Discussion

### Literature selection

**Figure 1.**
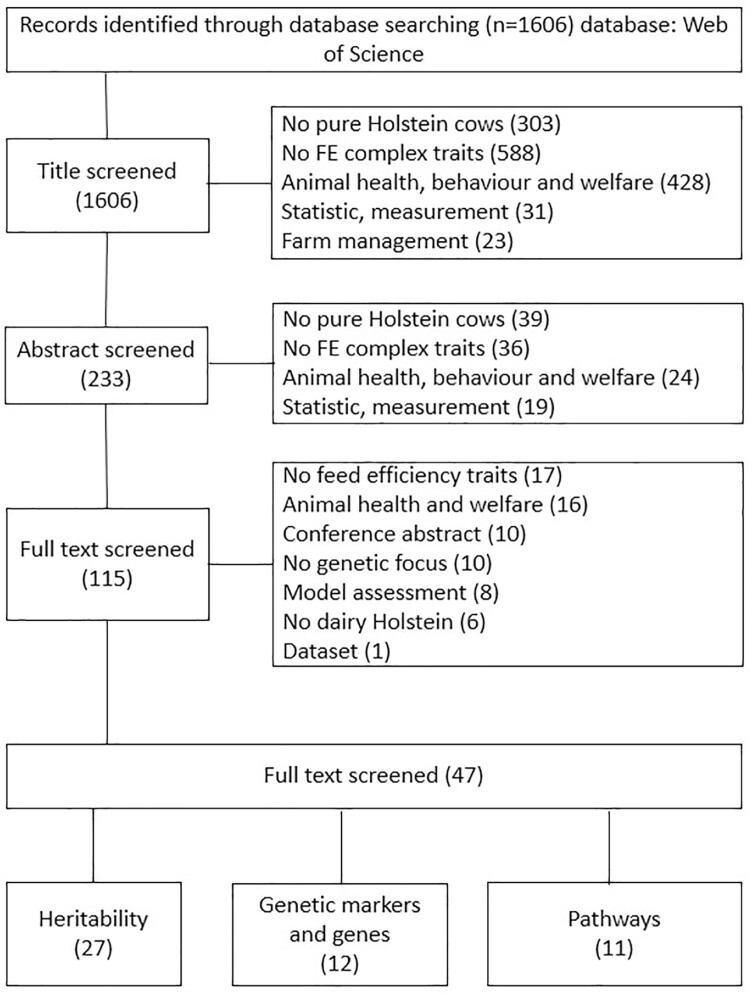
Selection process of literature returned through PRISMA search protocol. Note that the included categories were nonexclusive, with the same study where applicable included in multiple categories. (FE: feed efficiency).

A total of 1,606 articles were returned following the devised search protocol of PRISMA, with studies refined through three steps of screening for title, abstract, and full text, respectively (Figure 1). With this review only focused on pure Holstein breeds, the majority of articles were excluded during screening with studies on beefs, bulls, crossbreeds, other bovine breeds, and nonbovine species first removed. A number of studies were divided into “No FE complex traits” and then removed, including traits of body condition, nutrition, production, fertility, economic, emission, and rumen microbe. A large amount of research on bovine diseases, immune activities, and welfare were categorized into “Animal health, behavior, and welfare” and excluded as well. A total of 50 studies on statistical methods or measurement approaches of FE traits and 23 studies of farm management, such as diet suggestions of dry period cows or calves, additives, and feeding systems, were removed as well. Ten search results were conference abstracts, and together with one result which constituted a dataset, these were also removed. After the screening, 47 articles remained and were included in this review—27 articles for “heritability”, 12 for “genetic markers and genes”, and 11 for “pathway”. These articles were not mutually exclusive.

### Heritability estimates of FE traits and meta-analysis

The estimated heritability and number of phenotypic records for purebred Holstein reported in the literature are shown in Table 2. Studies using pedigree relationships for board-sense heritability estimates or genomic information matrices for narrow-sense heritability estimates were both included in this review. The heritability estimation ranges of RFI, DMI, EB, REI, and GFE from the literature are 0.01 to 0.40, 0.10 to 0.6, 0.03 to 0.52, 0.04 to 0.12, and 0.29 to 0.32, respectively. In addition to the comprehensive information obtained from the meta-analysis, an interesting finding is that FE complex traits share periodic characteristics. Bolormaa et al. (2022) reported a DMI heritability difference between Australian lactation cows (0.36 ± 0.09) and heifers (0.33 ± 0.09). Another study reported that heritability estimates of EB decrease as the parturition times increase (Ranaraja et al., 2018), which indicates EB may have similar genetic characteristics in primiparous and multiparous cows. Hardie et al. (2017) highlighted that the range of RFI phenotypes in parturient cows is wider than that in first-parturient cows. These findings imply that there may be some common genetic factors across different phenotypes that regulate animal FE and researchers should carefully consider the periodic impact when performing genetic parameter assessment.

**Table 2.** Heritability estimates and phenotype records of FE complex traits in Holstein dairy cows

Phenotype	Estimated heritability ± SE	Number of animals with phenotype	Reference
RFI			
P	0.15 ± 0.02	4,893	Tempelman et al. (2015)
G	0.14 ± 0.03 (primiparous)	3,075	Hardie et al. (2017)
G	0.13 ± 0.03 (multiparous)	2,667	Hardie et al. (2017)
G	0.27 ± 0.03 (international cows)	2,526	Bolormaa et al. (2022)
G	0.22 ± 0.07 (Australia Holstein)	1,000	Pryce et al. (2012)
G	0.38 ± 0.09 (New Zealand Holstein)	1,000	Pryce et al. (2012)
P	0.01 ± 0.05	970	Vallimont et al. (2011)
P	0.27 ± 0.12	903	Williams et al. (2011)
P	0.10 to 0.15	847	Islam et al. (2020)
G	0.33 ± 0.05	843	Gonzalez-Recio et al. (2014)
G	0.40 ± 0.09	842	Lin et al. (2013)
G	0.36 ± 0.09 (Australia heifers)	824	Bolormaa et al. (2022)
P	0.19 ± 0.05 (primiparous)	823	Li et al. (2017)
P	0.23 ± 0.05	650	Byskov et al. (2017)
G	0.19 ± 0.01 (Australian lactation cows)	584	Bolormaa et al. (2022)
P	0.11 ± 0.05	287	Connor et al. (2013)
P	0.20 ± 0.03	260	Manafiazar et al. (2016)
DMI			
G	0.12 to 0.53	8,737	de Haas et al. (2015)
G	0.34 ± 0.03	6,953	Berry et al. (2014a)
P, G	0.32 ± 0.03 (primiparous)	3,075	Hardie et al. (2017)
P, G	0.23 ± 0.03 (multiparous)	2,667	Hardie et al. (2017)
G	0.30 ± 0.03 (international cows)	2,526	Bolormaa et al. (2022)
G	0.58 ± 0.08 (primiparous)	1,804	Veerkamp et al. (2012)
G	0.26 to 0.37	1,174	Krattenmacher et al. (2019)
G	0.53 ± 0.08	970	Veerkamp et al. (2012)
P	0.17 ± 0.10	903	Williams et al. (2011)
G	0.44 ± 0.07	843	Gonzalez-Recio et al. (2014)
G	0.52 ± 0.03	842	Lin et al. (2013)
G	0.36 ± 0.09 (Australia heifers)	824	Bolormaa et al. (2022)
P	0.10 to 0.30	755	Berry et al. (2007)
P	0.37 ± 0.06	650	Byskov et al. (2017)
G	0.33 ± 0.09 (Australian lactation cows)	584	Bolormaa et al. (2022)
P	0.04 to 0.19	525	Buttchereit et al. (2011)
P	0.27 to 0.63	402	Spurlock et al. (2012)
P	0.28 ± 0.06	260	Manafiazar et al. (2016)
EB			
G	0.15 to 0.48	1,322	Harder et al. (2020)
P	0.06 to 0.18	1,274	Hurley et al. (2017)
G	0.29 to 0.49	1,174	Krattenmacher et al. (2019)
P	0.06 to 0.29	755	Berry et al. (2007)
P	0.03 to 0.13	525	Buttchereit et al. (2011)
G	0.33 ± 0.12	548	Verbyla et al. (2010)
P	0.19 to 0.52		Ranaraja et al. (2018)
REI			
P	0.12 to 0.39	1,341	Becker et al. (2021)
P	0.04 to 0.11	1,274	Hurley et al. (2017)
GFE			
P	0.32 ± 0.13	402	Spurlock et al. (2012)
P	0.29 ± 0.02	260	Manafiazar et al. (2016)

SE, standard error; RFI, residual feed intake; DMI, dry matter intake; EB, energy balance; REI, residual efficiency intake; GFE, gross feed efficiency; P, pedigree-based data; G, genome-based data.

**Figure 2.**
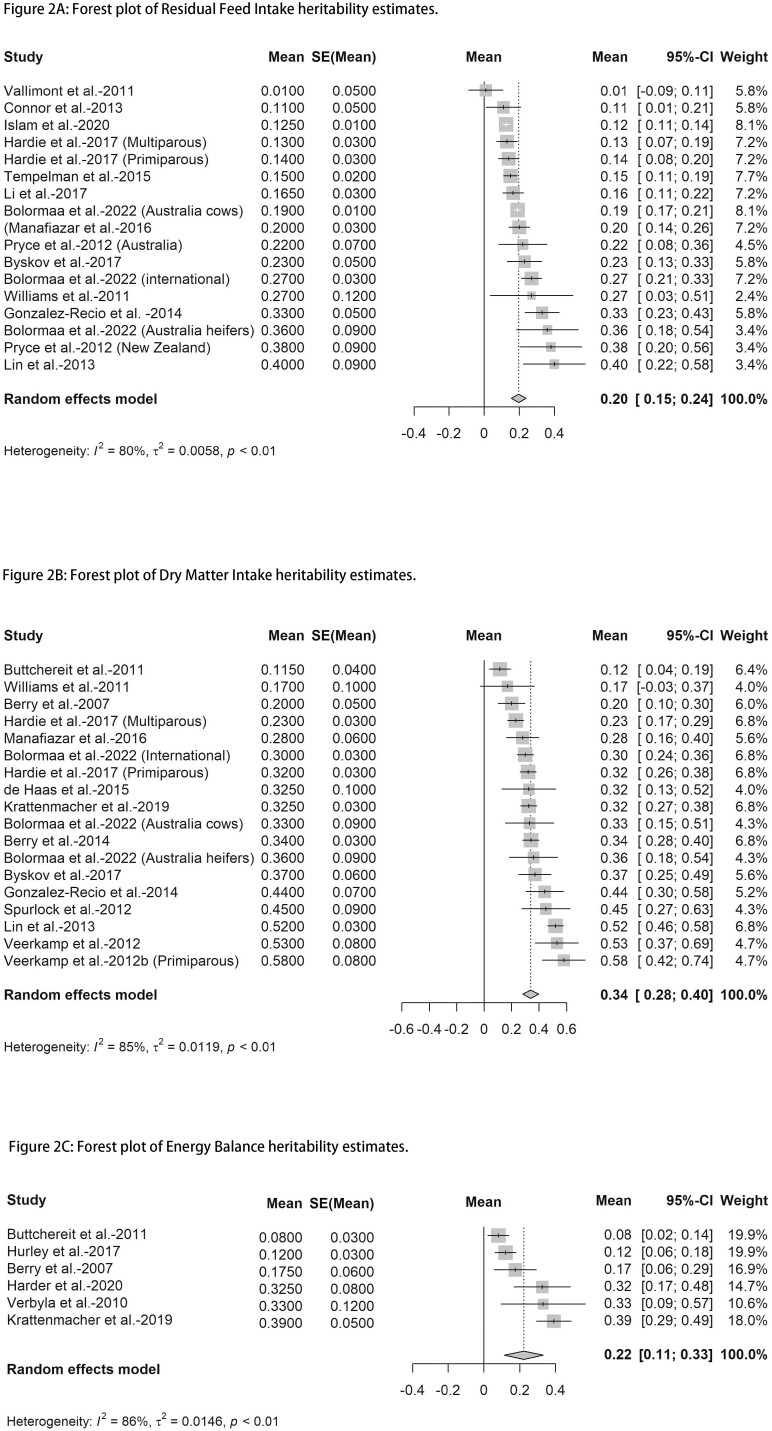
Forest plots of heritability estimate from meta-analysis of three FE phenotypes: (A) residual feed intake, (B) dry matter intake, (C) energy balance. Square size represents the weight of the study. The larger the square size indicate the more weight of the study on the mean effect size. The horizontal line represents a 95% CI. SE, standard error; CI, confidence interval; I^2^, heterogeneity between studies.

Meta-analysis was applied to a comparable number of studies to estimate pooled heritability for FE traits in Holstein dairy cows. The estimated heritability with 95% CI for RFI (17 studies), DMI (18 studies), and EB (6 studies) were found to range from 0.15 to 0.24, 0.28 to 0.40, and 0.11 to 0.33, respectively. Forest plots from performed meta-analysis (Figure 2A, B, and C) display the heritability estimates of RFI (0.20 ± 0.02), DMI (0.34 ± 0.03), and EB (0.22 ± 0.06) in meta-analysis, which implies genomic selection for these traits to improve FE is feasible in dairy cows. All three FE traits in meta-analysis (RFI, DMI, and EB) were observed to possess moderate heritability estimates (>0.20), which are well within acceptable boundaries for inclusion into breeding programs. It is worth noting that for GFE and REI, only two articles were included in each cluster, which is not sufficient to perform meta-analysis. Meta-analysis results showed high heterogeneity, which may be caused by many reasons (e.g., sample size, management strategy, genetic variation between animals, and animal characteristics differences between and within studies).

#### Dry matter intake

DMI is the amount of all feed consumed by a cow per day on a moisture-free basis. Feed intake is the basis for evaluating FE and is a key component of available FE indicators (Negussie et al., 2019). However, due to the high cost of measuring DMI in individual animals, DMI observations are mainly derived from research farm records, which are limited to large-scale genomic analysis. Most studies referred to DMI as a moderate heritable trait. DMI heritability ranged from 0.10 to 0.63 among all returned search results (Table 2). In earlier studies of DMI, estimates of heritability varied widely between studies, usually with a high estimated standard deviation (Buttchereit et al., 2011; Veerkamp et al., 2012). From screened studies, differences in DMI heritability estimates between studies can be attributed to four factors: 1) the use of genealogical relationships or genomic data, 2) differences in breeding systems in different countries, 3) physiological differences between primiparous and multiparous cows, and 4) population size of research animals. Berry et al. (2014a) firstly integrated data on individual daily feed intake of Holstein–Friesian cows and heifers in nine countries and reported the estimated a DMI heritability in the entire data set was 0.34 ± 0.03 when analyzed through the pedigree relationship matrix and 0.27 ± 0.02 when using the matrix combining pedigree and genomic relationship. For different countries, heritability ranged from 0.12 to 0.53. Considering the difference in rations, feeding systems, feed intake measurement techniques, recording methods, statistical models, and research animal populations, DMI heritability estimates could be different. For example, North America and Europe tend to use high input systems with high concentrate, while Australian cows are fed alfalfa chunks more and some concentrate during the milking period.

#### Residual feed intake

RFI as a proposed FE trait, is defined as the difference between the predicted feed intake and actual feed intake of individual animal, usually calculated from various energy sinks (Li et al., 2020). High RFI cows tend to have low FE while low RFI animals have high FE. RFI phenotypically does not correlate with animal size, weight gain, and milk production (Van Arendonk et al., 1991), and instead responds more to individual animal characteristics of metabolic and energy-use efficiency than production characteristics (Crews Jr., 2005). RFI heritability estimates ranged from 0.01 to 0.40 among 17 studies (Table 2). Among these publications, one study emphasized the lactation stage was an unneglected factor when assessing RFI (Li et al., 2017). Two models were compared in this study: model [1] used a 1-step RFI estimate model, and model [2] divided 44 lactation data into 11 consecutive lactation periods, each of 4 wk in length. RFI heritability in model [2] was proved more stable but slightly lower than that in model [1]. When reducing the length of data measurement, the variance from the environment would be reduced as well, considering the energy sinks differ dynamically during the entire lactation stage.

#### Energy balance

EB is defined as the difference between energy intake and energy expenditure of growth, maintenance, lactation, and fertility (Dorji et al., 2021). In early lactation, the elevated energy demand for milk production usually results in a negative energy balance (**NEB**) in most dairy cows, which may have a negative influence on present and future milk production (Churakov et al., 2021). A severe NEB is more likely to increase the risks of metabolic diseases (Pérez-Báez et al., 2019). Seven studies reported EB as a low to moderate trait which would likely respond to pressure, with heritability ranging from 0.03 to 0.52 (Table 2). EB is defined as the difference between energy intake and energy usage which occurs in the early lactation period, composed of milk production and composition, feed intake, and body weight (Veerkamp et al., 2000). An interesting finding is that heritability estimates of EB decrease as the parturition times increase (Ranaraja et al., 2018). EB may, like DMI, have genetic characteristics in primiparous and multiparous cows. However, due to the small number of studies in this area, further research is needed.

#### Gross feed efficiency

GFE is defined as the energy captured in the products divided by the total energy consumed over a cow’s lifetime (VandeHaar et al., 2016). Utilization of easy-to-measure traits (e.g., live weight, milk yield) to predict GFE via developed models has been widely considered (Dórea et al., 2018; Madilindi et al., 2022a). Among search returns, two studies reported the heritability of GFE, both estimates are moderate. Spurlock et al. (2012) reported heritability estimates for GFE were 0.47 ± 0.23 for 227 primiparous cows, 0.43 ± 0.25 for 175 multiparous cows, and 0.32 ± 0.13 for all 402 Holstein cows, data collected from 2 to 150 DIM (day in milk). Manafiazar et al. (2016) reported estimate for GFE was 0.29 ± 0.02, which is similar to Spurlock. Although GFE has a moderate heritability, it does not consider the energy flowing to body maintenance and growth, which may limit its ability to characterize high-feed efficient animals.

#### Residual efficiency intake

REI is defined as net energy intake minus predicted energy requirements, such as net lactation energy, maintenance, and body tissue anabolic metabolism (Olijhoek et al., 2020). REI is estimated in an approach similar to calculating EB and has a moderate to strong correlation with EB. Low REI animals (high-feed efficient) were usually in more severe negative EB, which is associated with disease tolerance. Two studies assessed the heritability of REI as a low to moderate trait, 0.12 to 0.39 in 1,341 cows (Becker et al., 2021), and 0.04 to 0.11 in 1,274 cows (Hurley et al., 2017), which indicates that it is feasible to directly select REI as FE index in future breeding goals, especially, within a holistic breeding goal, REI could be used to overcome the antagonisms between high-efficient animal and NEB.

### Genetic markers influencing FE in purebred Holstein

The reported genetic markers from studies with PDR-adjusted *P* value < 0.05 were tabulated in Table 3. These markers (Table 3) are considered statistically significant between herds and have the potential to be indicators for breeding programs.

**Table 3:** Descriptive genetic FE markers for FE complex traits from articles

Position	Genetic marker	Corresponding trait	Related gene	Reference
Chr 3, 14.9 Mb	CNV	RFI	Relaxin/insulin-like family peptide receptor 4 RXFP4	Zhou et al. (2018)
Chr 4, 108.2 Mb	CNV	DMI, RFI	OR2A2—Olfactory receptor gene	Zhou et al. (2018)
Chr 7, 42.7 Mb	CNV	RFI	OR2T12, OR2AK2—Olfactory receptor gene	Zhou et al. (2018)
Chr 14, 46.8Mb	SNP	RFI	FABP4 (fatty acid binding protein 4)	Cohen-Zinder et al. (2016)
Chr 15, 25.7 Mb	SNP	DMI, 80 DIM	HTR3B—5-hydroxytryptamine (serotonin) receptor 3B/3A	Tetens et al. (2014)
Chr 16, 11.2 Mb	SNP	DMI, 180 DIM		Tetens et al. (2014)
Chr 18, 57.7 to 58.2 Mb	SNP	DMI, RFI	VSIG10L (V-set and immunoglobulin domain containing 10 like)	Li et al. (2019)
Chr 25, 40.7 to 41.5 Mb	SNP	RFI	CARD11 (caspase recruitment domain family member 11)—protein-coding gene, EIF3B (Eukaryotic translation initiation factor 3 subunit B)	Li et al. (2019)
Chr 25, 14.1 Mb	SNP	DMI, 80 DIM	GDE1—glycerophosphodiester phosphodiesterase 1	Tetens et al. (2014)
Chr 27, 22.7 Mb	SNP	DMI, 11 DIM		Tetens et al. (2014)
Chr 28, 3.1 Mb	SNP	DMI, 30 DIM	Cluster of 23 olfactory receptor genes	Tetens et al. (2014)
Chr X, 119.4 Mb	SNP	DMI, 180 DIM		Tetens et al. (2014)
	miRNA	EB	LRP2 (low-density lipoprotein receptor-related protein 2)	Fatima and Morris (2013)

SNP, single-nucleotide polymorphism; CNV, copy number variance.

#### Single-nucleotide polymorphisms

SNP is the substitution of a single nucleotide at a certain position in the genome (Keats and Sherman, 2013). It can help to explain genetic differences in animal traits. SNP is a widely used molecular genetic marker in GWAS studies, which can provide FE-related gene regions on chromosomes. On the 40.7 to 41.5 Mb region of Chr 25, The strongest SNP effect of RFI was reported using high-density SNP chips (Li et al., 2019). This region overlaps with two genes, caspase recruitment domain family member 11 (CARD 11) and Eukaryotic translation initiation Factor 3 subunit B (EIF3B). CARD11 is a protein-coding gene in Bos taurus, which has been reported to be downregulated in cows with high RFI (Salleh et al., 2017). EIF3B is a protein-coding gene associated with protein synthesis initiation (Lee et al., 2015). The protein expression of EIF3B was increased in high FE broilers (Kong et al., 2016). Cohen-Zinder et al. (2016) reported a number of SNPs associated with RFI, particularly on Chr 14, where 10 SNPs overlap with fatty acid binding protein 4 (**FABP4**) regions (3ʹ-UTR, exons and promoters). FABP4 is a lipid-binding protein transporter that exists in fat cells controlling fatty acid uptake, transport, and metabolism (Snelling et al., 2010). FABP4 has been reported to be associated with lipid deposition in cattle and may be a significant candidate marker for lipid metabolism (Xu et al., 2011).

#### Copy number variance

Copy number variance (**CNV**) is an important type of mammal genetic variation that causes phenotypic differences (Bickhart and Liu, 2014). It has been widely considered as an alternative marker to SNP for GWAS (Gao et al., 2017; Zhou et al., 2018). Zhou et al. (2018) characterized three significant CNVs for phenotypes related to RFI or DMI (FDR-adjusted *P* value < 0.05). CNV 1 (Chr 4, 108.2 Mb) was shared by DMI and RFI, which overlaps olfactory receptor gene OR2A2 (LOC787786). Olfactory receptors may influence food preference and feeding activity, thus affecting RFI and DMI (Soria-Gomez et al., 2014). CNV2 (Chr 7, 42.7 Mb) overlaps with two olfactory receptors OR2T12 (LOC787816) and OR2AK2, which is another important CNV related to RFI. CNV 3 (Chr3 14.9 Mb) is located closely to relaxin/insulin-like family peptide receptor 4 (RXFX4). RXPX4 has been found to be associated with obesity in humans and has a signal transduction role in glucose metabolism (Kao and Müller, 2013; Yegorov et al., 2014). In addition, insulin-like peptide 5 (Insl5), a ligand for PXPX4, is produced by L cells in the gut and stimulates appetite to drive animals to forage when feed intake decreases (Grosse et al., 2014). In dairy cows, there may be similar foraging regulatory mechanisms that affect RFI.

#### MicroRNA

MicroRNA (miRNA) is one class of single-stranded noncoding RNA molecules that regulates many BPs at the posttranscriptional level, usually composed of 22 nucleotides in length (Xia et al., 2021). MicroRNA has been reported that can regulate the expression of up to 60% of protein-coding genes in mammals (Friedman et al., 2009). Many studies have reported that miRNA is associated with many important economic traits of livestock (Nicholas et al., 2013, Fatima and Morris, 2013) a previous study reported universally expressed and liver-specific miRNAs in beef cattle (Jin et al., 2009; Becker et al., 2011). Fatima et al (2014) reported a single miRNA—miR-143, which is differentially expressed between mild and severe NEB groups in liver tissues. RT-qPCR validation confirmed miR-143 was 2.4-fold downregulated in severe NEB group (FDR < 0.005). The author also predicted four potential genes as putative targets of miR-143. Low-density lipoprotein receptor-associated protein 2 (LRP2) is an interesting miR-143 target. LRP2 functions as a sterol receptor involved in lipid metabolism (Willnow et al., 2007), and there is evidence that lipid metabolism changes during NEB in dairy cows (Grummer, 1993).

### Reported candidate genes and pathway enrichment

A total of 169 genes from selected studies were included in a gene list (Supplementary Material 1). These genes came from three sources: differentially expressed genes between high/low FE animal groups, candidate genes associated with reported genetic markers, and mapped genes in the associated window. Among the gene list, the majority of genes were reported only once, except PEBP1, which was reported twice in different studies (Hou et al., 2012; Dorji et al., 2021). Dorji et al. (2021) conducted a differential expression analysis using the top and bottom 14 animals in RFI and EB ranking, whereas Hou et al. utilized Bovine HD SNP genotyping data came from extremely high or low estimated breeding value animals for CNV analysis. PEBP1 (Chr 17, 60 Mb) is a protein-coding gene, the participating in MAPK, ERK1/ERK2 pathways (Kyriakis and Avruch, 2012; Roskoski, 2012). The encoded protein can be further processed to form smaller cleavage products of Hippocampal cholinergic neurostimulating peptides and function as presynaptic cholinergic neurons in the central nervous system (Goumon et al., 2004). In bovine, PEBP1 mRNA in Holstein bulls’ serum was reported to be associated with fertility (Arangasamy et al., 2011).

GO analysis and KEGG analysis were performed on the gene list obtained from the literature to obtain extra and acceptable knowledge under a larger genome-scale condition. Among GO:BP analysis, there are three significant GO:BP terms ($P\;value<1.67\times 10^{-3}$), which are purine ribonucleoside triphosphate metabolic process, purine nucleoside triphosphate metabolic process, and ribonucleoside triphosphate metabolic process. Similarly, KEGG returns four significant terms (*P*-value < 8.84 × 10^−5^): diabetic cardiomyopathy, oxidative phosphorylation, chemical carcinogenesis (reactive oxygen species), and thermogenesis. Involved genes include ATP5F1D, ATP5MC2, ATP5MC3, MYH3, NME3, NME6, NME7, TSPO, COX17, COX7A2, GSTA3, MMP9, NDUFA1, NDUFA11, NDUFA4, NDUFB1, NDUFB11, PPP1CB, PRKCA, PTPN11, SP1, UQCR10, UQCRQ. The terms referring to genes were given in Supplementary Material 2. The meta-analysis result of 169 FE-related genes mainly focused on the biological mechanism of ATP synthesis, electron transport chain, and oxidative phosphorylation (OXPHOS) pathway. Among the involved genes, ATP5MC2, NDUFA, COX7A2, UQCR, and MMP are particularly important because they have also been reported in previous studies.

ATP5MC2 encodes a subunit of mitochondrial ATP synthase. It is involved in oxidative phosphorylation, using a transmembrane proton gradient caused by electron transport to convert to ATP (He et al., 2017). A recent study reported ATP5MC2 using GWAS approach and classified it as respiratory mitochondrial electron activity (Li et al., 2019). NDUFA 1 (NADH: Ubiquinone Oxidoreductase Subunit 1) Encodes an important part of the respiratory chain complex I, transferring electrons from NADH to ubiquinone (Stroud et al., 2016). Related genes encoding other subunits, e.g., NDUFAB1, NDUFA10, NDUFA8, NDUFA5, and NDUFA6, have been reported several times in the literature (Swartz et al., 2021, Dorji et al., 2021). COX7A2 (cytochrome c oxidase subunit 7A2-like) encodes a subunit of cytochrome c oxidase (**COX**). COX is a terminal component of the mitochondrial respiratory chain that catalyzes the transfer of electrons from reduced cytochrome c to oxygen (Lapuente-Brun et al., 2013). A study on the liver proteome of cows with NEB reported that COX7A2, COX7A2L, and COX6C may be associated with mitochondrial dysfunction, possibly resulting in NEB (Swartz et al., 2021). UQCR (ubiquinol–cytochrome c reductase complex) encodes a subunit of the panthenol–cytochrome c reductase complex, which constitutes part of the electron transport chain of oxidative phosphorylation. A recent proteome meta-analysis study reported that the protein UQCRC1 in milk is associated with mitochondrial oxidation, and this protein was proposed as a potential biomarker to identify NEB in dairy cows (Delosière et al., 2019). MMP9 (matrix metallopeptidase 9) encodes matrix metallopeptidase (MMP). MMP can degrade a variety of extracellular molecules and a variety of bioactive molecules. Wathes et al. (2011) reported that MMP1, MMP3, MMP9, and MMP13 were related to growth hormone–insulin-like growth factor (**GH–IGF**) axis uncouple. The expressions of MMP1, MMP3, MMP9, and MMP13 mRNA were significantly upregulated in the endometrium of SNEB dairy cows, and all of them except MMP9 were through interaction with IGFBP. MMP cleaves IGFBP1, IGFBP3, and IGFBP5, and increases the bioavailability of IGF to receptor activation (Nagase et al., 2006). An increase in MMP3 has been reported to be directly associated with bovine endometritis and its increase may contribute to the progression of bovine endometritis (Zhang et al., 2021). The above genes participate in respiratory electron transport, ATP synthesis, OXPHOS pathway, and IGF axis, and are associated with at least one type of FE complex. Considering the pathway enrichment results derived from a gene list use a more stringent cutoff value, the above genes and biological mechanisms could more accurately reflect the biological mechanisms affecting FE in dairy cows. These genes may provide a potential new target for manipulating traits related to FE in livestock.

### Other reported pathways associated with FE complex in literature

The reported biological pathways and genes associated with FE from selected studies are displayed in Supplementary Material 3. The summarized pathway terms and genes overlap to some extent with the results of previous pathway enrichment. According to 11 “Pathway” studies, at least one of transcriptomic, proteomics, metabolomics, and differential expression analysis methods has been applied to specifically discover biological mechanisms. The findings could be roughly divided into four representative parts according to biological functions (IGF axis, oxidative phosphorylation, lipid metabolism, and amino acid metabolism).

#### Insulin-like growth factor axis

IGFs are proteins sharing highly similar sequences with insulin. IGFs are part of the IGF axis that allows cells to communicate with the external physiological environment and play an important role in cell proliferation and death (Vasques et al., 2019). Four studies (Wathes et al., 2011, Veerkamp et al., 2012, Xi et al., 2015, Wang et al., 2020) have reported that IGF axis may play an important role in the feeding efficiency of Holstein dairy cows, especially NEB in early lactation. Increased expression of IGF-binding protein 4 (IGFBP4) mRNA was detected in endometrium of cows with severe NEB, with increased expression of IGFBP1 and decreased expression of IGFBP-6 (Wathes et al., 2011). IGFBP4 binds IGF1 and IGF2 and is usually co-expressed with IGF2 during development (Ning et al., 2008). In an early study, decreased IGFBP-2 and IGFBP-6 expression was reported in the fallopian tubes of NEB cows in a previous study (Fenwick et al., 2008). Postpartum uterine IGFBP regulates the function of IGF, and the differential expression of IGFBP is based on the energy state of lactating cows and has tissue specificity. In a recent study (Wang et al., 2020), adding dietary rumen-protected glucose leads to the upregulation of IGF in the endometrium of cows in the early postpartum period to promote the proliferation of endometrial cells. However, the role of IGFBP and the precise mechanism controlling their expression in the endometrium remain to be elucidated.

Insulin is thought to be another key factor in regulating the postpartum IGF system in utero (Wathes et al., 2011). Usually in late pregnancy, insulin decline stimulates fat mobilization, which provides nutrients to the fetus and leads to an increase in circulating nonesterified fatty acids (**NEFA**) concentrations (Zinicola and Bicalho, 2019). NEB cows were detected to have higher cyclic NEFA and lower IGF1 after paring, with possible peripheral insulin resistance. This study also detected high expression of alpha 2-hs-glycoprotein (AHSG) mRNA in the uterus of SNEB dairy cows, which was positively correlated with circulating NEFA concentration. AHSG, a plasma protein produced by the liver, is positively associated with human insulin resistance, supporting the existence of peripheral insulin resistance in dairy cows during NEB (Sivan and Boden, 2003). Xi et al. (2015) found high expressions of IRS and AKT1 in cows with high RFI and speculated that insulin signaling might be related to RFI. Veerkamp et al., 2012 reported the candidate gene affecting FE complex trait is also related to insulin, consistent with Wathes’s conclusion.

#### Oxidative phosphorylation

Oxidative phosphorylation is a common metabolic pathway that occurs in eukaryotic mitochondria to release energy through the oxidation of nutrients by various enzymes (Tang et al., 2020). Two studies reported that OXPHOS are associated with EB. Dorji et al. (2021) found that mitochondrial protein genes were under-expressed in the more feed-efficient group, which may indicate lower metabolic turnover, resulting in less energy production and heat loss. This study also demonstrated that mitochondrial protein genes were differentially expressed in different FE groups through blood transcriptome studies, especially COX4I1. COX4I1 is involved in encoding ETC complex IV in mitochondria (Yoshikawa, 1997). In humans, mutations in COX4I1 lead to short stature and difficulty gaining weight (Abu-Libdeh et al., 2017). More research is needed to elucidate the biological mechanism of COX4I1 in dairy cows.

Swartz et al. (2021) performed a differential analysis of liver proteome and identified proteins with differential abundance such as NDUFA5, NDUFS3, COX7A2L, and NDUFA6. The proteins NDUFS3, NDUFA5, and NDUFA6 are subunits of NADH dehydrogenase; COX7A2L is a subunit of cytochrome c oxidase (Ernster and Schatz, 1981). These proteins participate in the mitochondrial respiratory chain on the inner membrane of the mitochondria as part of OXPHOS. In summary, differential protein expression in the liver of NEB dairy cows indicates low liver energy production efficiency and mitochondrial dysfunction, which possibly leads to efficient cows relying more on adipose tissue mobilization as an energy source after pregnancy and are more prone to excessive weight loss. In Nellore bulls with high RFI, OXPHOS-related genes were reported to be highly expressed (Benedeti et al., 2018).

#### Lipid metabolism

Adipose tissue, as the main energy reserve of mammals, plays a crucial role in milk production in dairy cows. Ben Meir et al. (2022) found that transferrin is a differentially expressed protein in mid-lactation cows through proteomics, which is upregulated in eight Fe-related pathways and can be used as a candidate biomarker. Three SNPs of transferrin were detected in a recent study (Ali et al., 2022).

One study reported that the abundance of angiopoietin-like protein 4 (ANGPTL4) mRNA was increased in all three models of declining EB, suggesting that ANGPTL4 may act as an early autocrine/paracrine or endocrine signal to influence energy status in lactating cattle (Koltes and Spurlock, 2012). Kong et al. (2020) reported that ANGPTL4 promotes the transport of triglycerides in the form of very low-density lipoprotein, contributing to the adaptive regulation of the lipid transport system in dairy cattle. Some studies have reported that ANGPTL4 is associated with perinatal fatty liver, ketosis, and other metabolic disorders in dairy cows (Wang et al., 2018; Kong et al., 2020).

Swartz et al. (2021) analyzed the differential expression gene profiles of jugular vein serum of cows with different RFI groups and found that the adipocyte signaling pathway affected FE characters of cows. Leptin (LEP), an adipocyte-derived protein from the obesity gene, is a key factor in this pathway (Swartz et al., 2021). The plasma concentration of leptin is positively correlated with fat volume, which regulates anorexia in animals (Collins et al., 1996). Differential expression of LEP mRNA between extreme RFI groups in Angus cattle has been reported (Perkins et al., 2014). Swartz et al. (2021) also reported that beta-enolase (ENO3) and fatty acid binding protein 5 (**FABP5**) are differentially expressed proteins based on NEB dairy cow liver proteomics. ENO3 is an enzyme that mediates cholesterol ester synthesis, affects lipid accumulation in the liver, and is one of the markers of human obesity (Koltes and Spurlock, 2012; Jia and Zhai, 2019). FABP5 was reported as an intracellular fatty acid transporter that directs fatty acids to appropriate locations for various cellular processes (e.g., oxidation) that regulate lipid metabolism and inflammatory responses (Furuhashi and Hotamisligil, 2008). However, more research is needed to explain the possible function of these proteins in cow fatty liver and ketosis.

#### Amino acid metabolism

Amino acids are the basis of protein synthesis, the substrates for many biosynthesis (e.g., ATP, fatty acids), and the precursors of many biological molecules (e.g., signaling molecules, nucleic acid bases) (Chandel, 2021). Amino acid metabolism is important for epigenetic modifications (e.g., DNA methylation, RNA methylation). The metabolism pathway of tryptophan was different in different FE dairy cows, and the concentrations of tryptophan and its main metabolite kynurenine decreased in high-efficiency dairy cows. Tryptophan (95%) is degraded by the kynurenine pathway in the liver (Wu, 2021). Martin et al., 2021 utilized untargeted metabolomics for broader research and identified seven amino acids that differed among FE cows, which may also support differences in tissue preference for amino acid utilization. A recent study proposes that adaptive responses alter the purpose of amino acid absorption and the efficiency with which these amino acids are ultimately converted into milk (Souzaa et al., 2023).

IDO2 has been proposed as a candidate gene for DMI based on the discovery of related SNPS on Chr 27 (Veerkamp et al., 2012). IDO2 gene plays an important role in tryptophan metabolism. As a direct precursor of serotonergic activity in the brain, tryptophan is associated with the regulation of dietary intake (Koopmans et al., 2006).

## Conclusions

A thorough understanding of the genetic factors that influence the FE of dairy cows is a prerequisite for planning and implementing selective breeding programs. Integrating existing information on genomic of FE could help researchers to better understand the biological background of FE. The utilization of random-effects models to perform a meta-analysis of animal genetic parameter estimation is a reliable method that overcomes the issues of insufficient study number and population size to a certain extent. Through meta-analysis, the heritability estimates of RFI, DMI, and EB were determined to be 0.22 ± 0.02, 0.34 ± 0.03, and 0.24 ± 0.06, respectively. The meta-analysis results of heritability estimation imply that it is feasible to incorporate FE traits into selective breeding. Although a large number of identified genetic markers have been reported in the literature, due to differences in environmental factors, research methods, herd management, and statistical methods, most SNP markers only show statistical significance for the single herd in the study. The significant genes reported in the literature were summarized, and a gene set containing 169 genes was obtained. Then, a pathway enrichment was carried out on the gene set, and the enrichment analysis results were mainly concentrated on energy metabolism and the respiratory chain. The pathway enrichment results overlapped with the biological mechanisms reported in the literature, such as OXPHOS pathway and gene ATP5MC2, NDUFA, COX7A2, UQCR, and MMP. Involved candidate genes, potential biomarkers, and proposed biological mechanisms from the literature are explained mainly in four biological mechanisms (IGF axis, lipid metabolism, OXPHOS, and tryptophan metabolism). Since this review is based on the FDR-adjusted *P* value as screening criteria for genetic markers, genes, and biomarkers statistically significant between herds, there may be omissions, that is, functional genetic factors in the single herd due to insufficient sample data or environmental factors did not show data significance. Therefore, it is necessary to increase the genomic test size to acquire more accurate genetic parameter estimation and integrate omics analysis to reveal underlying biological mechanisms.

## Supplementary Material

skae040_suppl_Supplementary_Data_S1

skae040_suppl_Supplementary_Data_S2

skae040_suppl_Supplementary_Data_S3

## Abbreviations

FE: feed efficiency
DMI: dry matter intake
EB: energy balance
RFI: residual feed intake
GFE: gross feed efficiency
REI: residual efficiency intake
BW: body weight
FDR: false discovery rate
SNPs: single-nucleotide polymorphisms
CNV: copy number variance
GWAS: genome-wide association studies
PRISMA: preferred reporting items for the systematic reviews and meta-analyses
PEO: population, exposure and outcome
χ2: Chi-square
NEB: negative energy balance
DIM: day in milk
OXPHOS: oxidative phosphorylation pathways
